# Killing two birds with one stone: dual blockade of integrin and FGF signaling through targeting syndecan-4 in postoperative capsular opacification

**DOI:** 10.1038/cddis.2017.315

**Published:** 2017-07-13

**Authors:** Yingyan Qin, Yi Zhu, Furong Luo, Chuan Chen, Xiaoyun Chen, Mingxing Wu

**Affiliations:** 1 State key Laboratory of Ophthalmology, Zhongshan Ophthalmic Center, Sun Yat-sen University, Guangzhou 510060, China; 2 Department of Molecular and Cellular Pharmacology, University of Miami Miller School of Medicine, Miami, FL 33136, USA

## Abstract

The most common complication after cataract surgery is postoperative capsular opacification, which includes anterior capsular opacification (ACO) and posterior capsular opacification (PCO). Increased adhesion of lens epithelial cells (LECs) to the intraocular lens material surface promotes ACO formation, whereas proliferation and migration of LECs to the posterior capsule lead to the development of PCO. Cell adhesion is mainly mediated by the binding of integrin to extracellular matrix proteins, while cell proliferation and migration are regulated by fibroblast growth factor (FGF). Syndecan-4 (SDC-4) is a co-receptor for both integrin and FGF signaling pathways. Therefore, SDC-4 may be an ideal therapeutic target for the prevention and treatment of postoperative capsular opacification. However, how SDC-4 contributes to FGF-mediated proliferation, migration, and integrin-mediated adhesion of LECs is unclear. Here, we found that downregulation of SDC-4 inhibited FGF signaling through the blockade of ERK1/2 and PI3K/Akt/mTOR activation, thus suppressing cell proliferation and migration. In addition, downregulation of SDC-4 suppressed integrin-mediated cell adhesion through inhibiting focal adhesion kinase (FAK) phosphorylation. Moreover, SDC-4 knockout mice exhibited normal lens morphology, but had significantly reduced capsular opacification after injury. Finally, SDC-4 expression level was increased in the anterior capsule LECs of age-related cataract patients. Taken together, we for the first time characterized the key regulatory role of SDC-4 in FGF and integrin signaling in human LECs, and provided the basis for future pharmacological interventions of capsular opacification.

Cataract remains the leading cause of vision loss worldwide, accounting for 11 million cases of blindness and 35 million cases of visual impairment every year.^1, 2^ Currently, phacoemulsification with intraocular lens (IOL) implantation is the most effective treatment for cataract. However, this procedure has many postoperative complications.^3, 4^ The most common complication after cataract surgery is postoperative capsular opacification, which includes anterior capsular opacification (ACO) and posterior capsular opacification (PCO). Both ACO and PCO can greatly affect the visual quality of cataract patients. ACO usually develops faster than PCO and may lead to IOL decentration, anterior capsule shrinkage, or hinder the examination of the peripheral fundus.^5^ PCO is more common than ACO, with an incidence of 20% to 40% in adult patients and 100% in children.^6^ Unlike ACO, PCO can involve the central visual axis, and it may, therefore, severely compromise visual acuity. Currently, the only effective treatment for PCO is Nd:YAG laser capsulotomy, which may induce further complications.

As lens epithelial cells (LECs) are distributed only underneath the anterior capsule, ACO and PCO have distinct pathogenic mechanisms. ACO is initially triggered by the adhesion of residual LECs and inflammatory cells in the aqueous humor onto the intraocular lens (IOL) surface and the subsequent proliferation and epithelial–mesenchymal transition (EMT) of LECs.^7^ In contrast, the first step of PCO is the migration of LECs from the anterior capsule to the posterior capsule.^6^ In the past decades, many attempts have been made to reduce ACO and PCO. One effective approach to reduce ACO is inhibiting the adhesion of cells onto the IOL surface. Notably, among all IOL materials, the most widely used hydrophobic acrylic IOLs have the highest incidence of ACO formation, as protein and cells can easily adhere to hydrophobic surfaces.^8^ Studies from our group and others have reported that hydrophilic surface modification of IOLs can significantly increase protein and cell repellence, thus preventing ACO.^9, 10^ On the other hand, PCO can be suppressed through blocking the migration and proliferation of LECs, which can be achieved by applying cytotoxic drugs or sharpening the optic edge of the IOLs.^11^ However, preventative approaches of ACO and PCO are contradictory. A reduction in LEC adhesion force may promote LEC proliferation and migration, resulting in increased risk of PCO development.^12, 13^

Here, we attempted to identify a key regulator in the pathogenesis of capsular opacification that suppresses both ACO and PCO when targeted. Cell adhesion is mainly mediated by integrin signaling. Shortly after IOL implantation, extracellular matrix (ECM) proteins, such as fibronectin, laminin, and collagen, will adhere onto the IOL surface. ECM proteins contain an Arg-Gly-Asp (RGD) sequence that can be recognized by integrin, which recruits and activates focal adhesion kinase (FAK), initiating cell adhesion.^14^ In contrast, cell migration and proliferation are promoted by various cytokines and growth factors.^15^ Fibroblast growth factor (FGF) is one of the most potent growth factors that regulates cell migration and proliferation. The FGF family comprises two prototypic members, acidic FGF (aFGF) and basic FGF (bFGF), as well as 21 related polypeptide growth factors.^16^ Among them, bFGF is considered as the most important regulator during LECs proliferation and differentiation, and is significantly upregulated in aqueous humor after cataract surgery.^17^ Mutations of the FGF receptor (FGFR) lead to aberrant cell growth and differentiation, resulting in developmental defects.^18, 19^ Apparently, an ideal approach is to simultaneously inhibit FGF-mediated LEC proliferation and migration, as well as integrin-mediated cell adhesion. Intriguingly, integrin signaling and FGF signaling share a common co-receptor, syndecan-4 (SDC-4).^20^ SDC-4 is a transmembrane proteoglycan that is highly expressed on epithelial cells.^21^ Inhibition of SDC-4 expression significantly delayed wound healing through suppression of cell proliferation and migration.^22^ In contrast, SDC-4 overexpression enhanced wound closure in diabetic mice.^23^ In addition, SDC-4 is a widespread component of focal adhesion, and focal adhesion formation is increased upon SDC-4 overexpression.^24, 25^ However, the role of SDC-4 in the proliferation, migration, and adhesion of LECs has not been fully elucidated.

Based on the essential co-regulatory role of SDC-4 in both FGF and integrin signaling, we hypothesize that targeting SDC-4 may effectively inhibit FGF and integrin signaling simultaneously, thus suppressing the formation of capsular opacification. In this study, we applied both *in vitro* and *in vivo* models to characterize the function of SDC-4 in the FGF and integrin signaling pathways in human LECs. Our study supported SDC-4 as a potential pharmacological target for preventative strategies against postoperative capsular opacification.

## Results

### SDC-4 downregulation suppressed FGF-induced cell proliferation through inhibition of the G1/S transition

**Figure 1:**
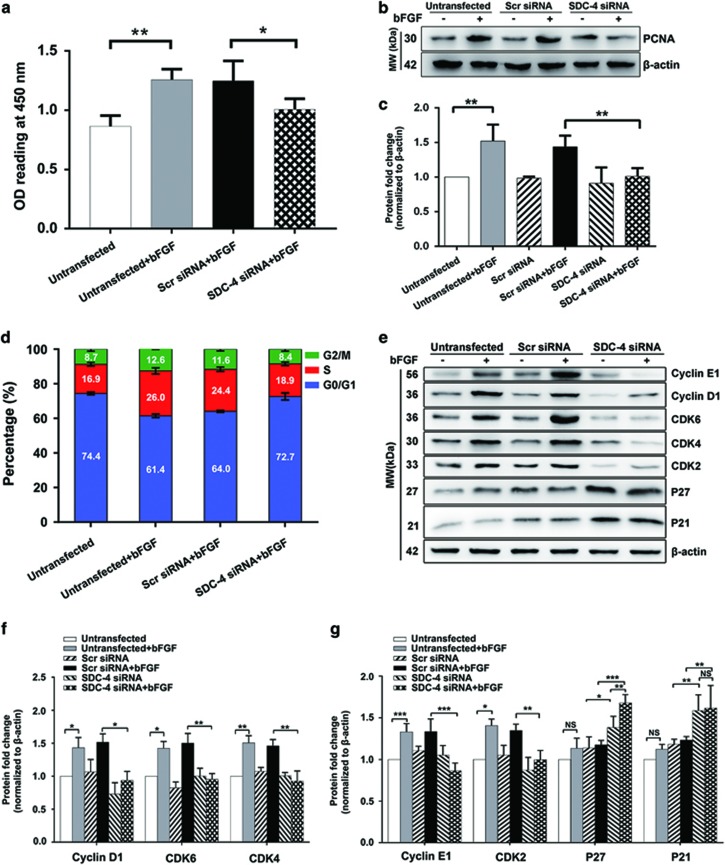
Downregulation of SDC-4 suppressed FGF-induced cell proliferation. (**a**) LECs were transfected with scrambled/SDC-4 siRNA for 48 h. Cell proliferation was analyzed by CCK-8 assay after 50 ng/ml bFGF treatment for 48 h. Data represent the mean±S.D. *P<0.05, **P<0.01, *n*=3. (**b**) LECs were transfected with scrambled/SDC-4 siRNA and treated with/without 50 ng/ml bFGF for 48 h. Western blot analysis was performed to probe for PCNA (30 kDa). *β*-actin (42 kDa) was used as an internal control. (**c**) Quantification of the PCNA expression level. The fold change relative to the level in the untransfected group is displayed. Data represent the mean±S.D. **P<0.01, *n*=3. (**d**) LECs were transfected with scrambled/SDC-4 siRNA and treated with/without 50 ng/ml bFGF for 48 h. Cell cycle analysis was performed by flow cytometry. (**e**) LECs were transfected with scrambled/SDC-4 siRNA and treated with/without 50 ng/ml bFGF for 48 h. Western blot analysis was performed to probe for cyclin E1 (56 kDa), cyclin D1 (36 kDa), CDK6 (36 kDa), CDK4 (30 kDa), CDK2 (33 kDa), P27 (27 kDa), and P21 (21 kDa). (**f** and **g**) Quantification of the protein expression levels in **e**. The fold change relative to the level in the untransfected group is displayed. Data represent the mean±S.D. *P<0.05, **P<0.01, ***P<0.001; NS, not significant, *n*=3

To determine the role of SDC-4 in FGF signaling, we knocked down SDC-4 in LECs by using RNAi as previously described.^26^ We observed a significant downregulation of SDC-4 after SDC-4 siRNA transfection at both transcript and protein levels (Supplementary Figure 1). Then, we performed a CCK-8 assay to evaluate cell proliferation. It has been shown that bFGF is a potent inducer for LEC proliferation.^27^ Indeed, we found that 50 ng/ml bFGF promoted LEC proliferation, as the group treated with bFGF for 48 h had significantly higher optical density (OD) values than that of the untreated group (1.257±0.091 *versus* 0.863±0.091, *P*<0.01). SDC-4 knockdown (KD) suppressed the proliferative effect of bFGF, as the OD value of the SDC-4 siRNA-transfected group was significantly lower than that of the scrambled siRNA-transfected group after bFGF treatment for 48 h (1.007±0.091 *versus* 1.247±0.170, *P*<0.05; Figure 1a), with an inhibition rate of 19.2%. We also normalized the OD value at 48 h to the OD value at 12 h to minimize the confounding factor of cell adhesion, and obtained comparable results (Supplementary Figure 2). Proliferating cell nuclear antigen (PCNA) is a cellular marker for proliferation.^28^ Consistently, we found that bFGF treatment for 48 h significantly upregulated the expression level of PCNA, which could be suppressed by SDC-4 KD (Figures 1b and c).

Inhibition of cell proliferation may result from an induction of apoptosis, suppression of cell cycle progression, or a combinatorial effect of both.^29^ We first assessed apoptosis by measuring the activation of caspase-3 and caspase-9. No significant differences in the levels of caspase-3, caspase-9, or their respective cleaved products were observed after SDC-4 KD, suggesting that the reduction in cell viability after SDC-4 KD was not due to increased apoptosis (Supplementary Figure 3). Next, we evaluated the cell cycle distribution by flow cytometry. We found that bFGF increased the percentage of cells in the S phase and reduced the percentage of cells in the G0/G1 phase. This bFGF-induced cell cycle progression can be suppressed by SDC-4 KD (Figure 1d). To further dissect how SDC-4 KD inhibited cells from entering into the S phase, we evaluated the expression levels of key cell cycle regulators. The G1/S transition requires the phosphorylation of retinoblastoma protein (Rb), which is initiated by the cyclin D1-cyclin-dependent kinase (CDK)6/CDK4 complex and completed by the cyclin E1–CDK2 complex.^30^ We found that treatment with 50 ng/ml bFGF for 48 h significantly increased the levels of cyclin E1, cyclin D1, CDK6, CDK4, and CDK2, which could be suppressed by SDC-4 KD (Figures 1e–g). In addition, SDC-4 KD upregulated the expression levels of P21 and P27 (Figures 1e and g), which act as CDK inhibitors and prevent the activation of cyclin E1–CDK2.^31^ We repeated these experiments in another well-established human lens epithelial cell line HLE-B3 and a human retinal pigment epithelial cell line ARPE-19, and obtained similar results(Supplementary Figures 4 and 5). Taken together, these data demonstrated that SDC-4 is required for the bFGF-promoted cell cycle progression.

### SDC-4 downregulation inhibited FGF signaling through blocking downstream activation of PKC*α*, ERK1/2, and PI3K/Akt/mTOR

**Figure 2:**
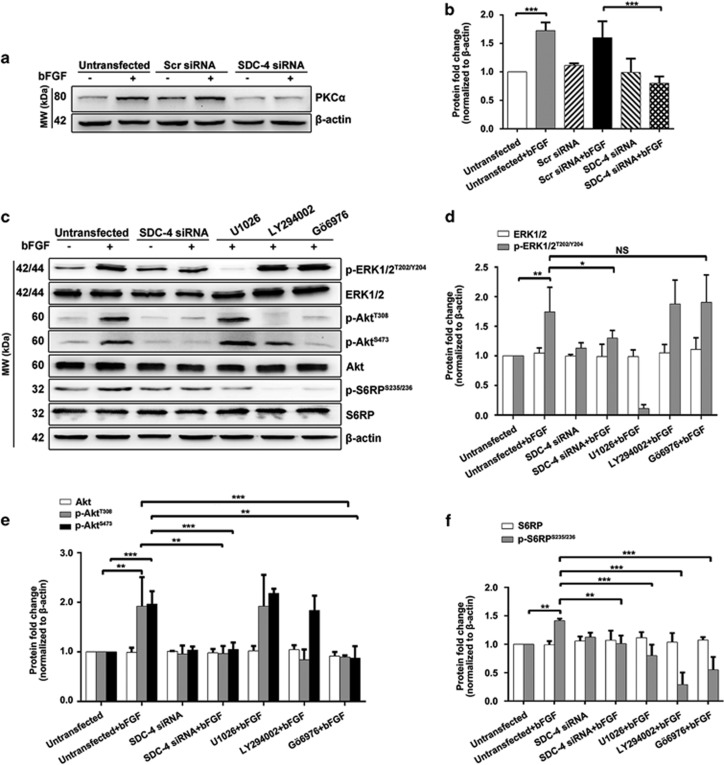
Downregulation of SDC-4 suppressed expression of PKC*α* and inhibited activation of the ERK1/2 and PI3K/Akt/mTOR pathways in LECs. (**a**) LECs were transfected with scrambled/SDC-4 siRNA and treated with/without 50 ng/ml bFGF for 48 h. Western blot analysis was performed to probe for PKC*α* (80 kDa). (**b**) Quantification of the protein expression levels in **a**. The fold change relative to the level in the untransfected group is displayed. Data represent the mean±S.D. ***P<0.001, *n*=3. (**c**) LECs were transfected with SDC-4 siRNA and treated with/without 50 ng/ml bFGF for 48 h, or LECs were pre-treated with U1026 (ERK1/2 inhibitor), LY294002 (PI3K inhibitor), or Gö6976 (PKC inhibitor) for 30 min and treated with 50 ng/ml bFGF for 15 min. Western blot analysis was performed to probe for p-ERK1/2 (42/44 kDa), total ERK (42/44 kDa), p-Akt (60 kDa), total Akt (60 kDa), p-S6RP (32 kDa), and total S6RP (32 kDa). (**d**–**f**) Quantification of the protein expression levels in **c**. The fold change relative to the level in the untransfected group is displayed. Data represent the mean±S.D. *P<0.05, **P<0.01, ***P<0.001; NS, not significant, *n*=3

FGF promotes cell proliferation mainly through the extracellular signal-regulated kinase 1/2 (ERK1/2) pathway and the phosphatidylinositol 3-kinase (PI3K)/Akt/mammalian target of rapamycin (mTOR) pathway.^32^ Phosphorylation of Akt leads to reduced expression of P21 and P27,^33, 34^ whereas phosphorylation of ERK1/2 and S6 ribosomal protein (S6RP, a downstream effector of mTOR) increases expression of the cyclin D1-CDK6/CDK4 complex.^35^ Dephosphorylation of the SDC-4 cytoplasmic tail has been shown to increase the binding of phosphatidylinositol 4, 5-bisphosphate PIP_2_ to SDC-4 and subsequently upregulate and activate protein kinase C*α* (PKC*α*), which is a potent activator of ERK1/2 and Akt.^20^ Indeed, we found that bFGF significantly upregulated the expression level of PKC*α*, and this upregulation required SDC-4 (Figures 2a and b). In addition, SDC-4 KD abrogated bFGF-induced ERK1/2, Akt, and S6RP phosphorylation (Figures 2c–f). Notably, we found that Gö6976, a PKC inhibitor, specifically suppressed phosphorylation of Akt and S6RP but had no inhibitory effect on phosphorylation of ERK1/2. Therefore, in LECs, PKC*α* is required for the activation of PI3K/Akt/mTOR but is dispensable for the activation of ERK1/2, and SDC-4 KD inhibited ERK1/2 phosphorylation through mechanisms independent of PKC*α*. In addition, U1026, an ERK1/2 inhibitor, suppressed bFGF-induced S6RP phosphorylation, which is consistent with the previous finding that mTOR is a common downstream target of ERK1/2 and Akt.^36^

### SDC-4 downregulation suppressed FGF-induced LEC migration

**Figure 3:**
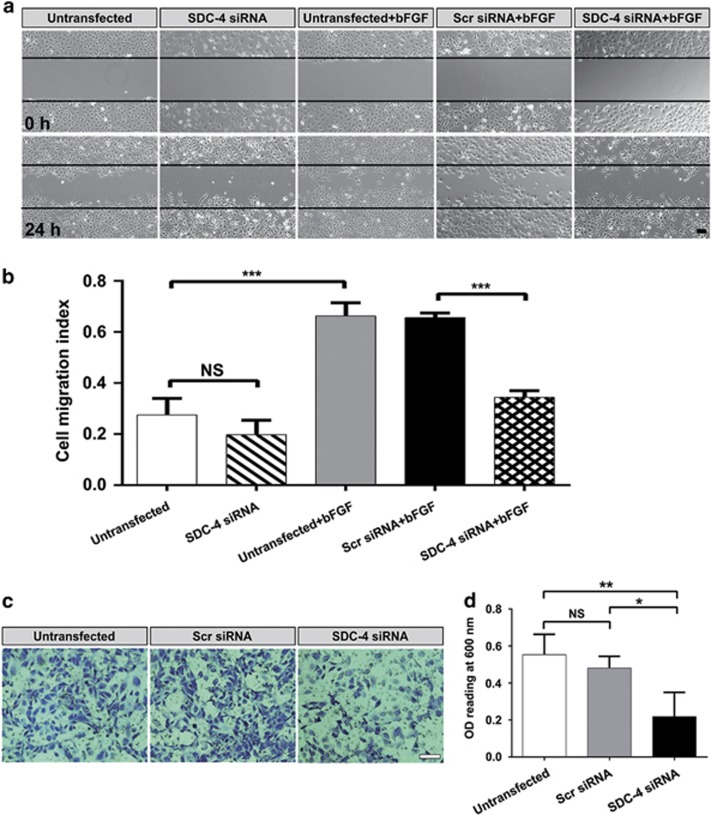
Downregulation of SDC-4 suppressed FGF-induced cell migration. (**a**) LECs were transfected with scrambled/SDC-4 siRNA and treated with/without 10 ng/ml bFGF for 24 h. Cell migration was observed by using an inverted phase contrast microscope. Straight black lines indicate the wound edges. Scale bar, 100 *μ*m. (**b**) Quantification of cell migration into the wound area. Data represent the mean±S.D. ***P<0.001, NS, not significant, *n*=3. (**c**) Representative images of LECs migrated to the lower chamber of the transwell filter. Scale bar, 100 *μ*m. (**d**) Quantification of the migrated LECs. Cells stained with crystal violet were solubilized with acetic acid. The absorbance (OD=600 nm) was measured in a microplate reader. *P<0.05, **P<0.01; NS, not significant, *n*=3

The migratory and proliferative effects of FGF have distinct pathways. FGF promotes cell migration through the activation of Src kinase and p38 mitogen-activated protein kinase (MAPK) rather than ERK or Akt.^37^ We next tested whether downregulation of SDC-4 also abrogated the migratory effects of FGF on LECs by the wound healing assay.^26^ We found that bFGF significantly increased LEC migration, which could be suppressed by SDC-4 KD (Figures 3a and b). In addition, the transwell assay showed that SDC-4 KD significantly suppressed cell migration towards the FGF-containing medium (Figures 3c and d). Consistent with our findings, previous studies have shown that overexpression of SDC-4 increased the motility of keratinocytes and fibroblasts,^38^ and SDC-4 knockout (KO) in mice resulted in impaired wound repair and mesenchymal cell migration.^39, 40^

### SDC-4 downregulation suppressed integrin-mediated LEC adhesion

**Figure 4:**
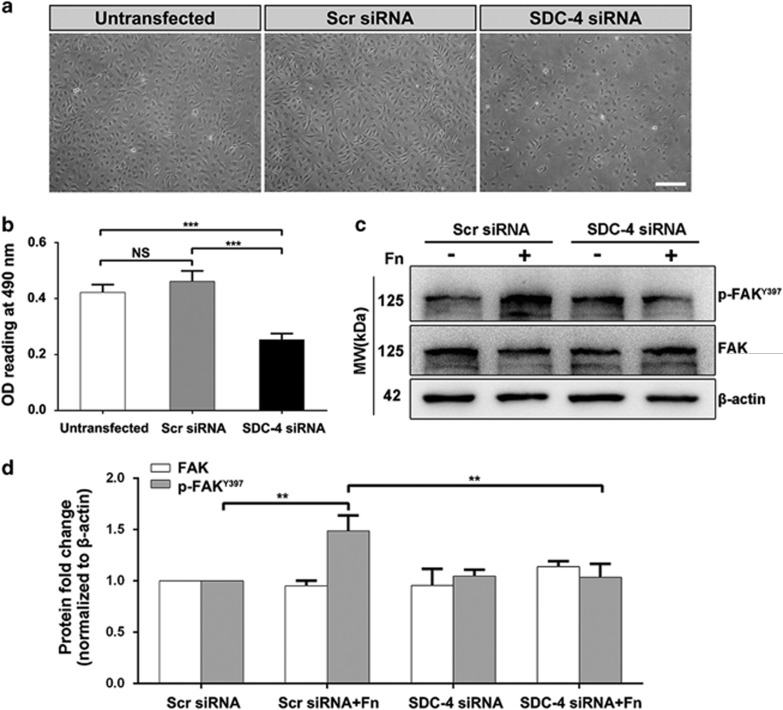
Downregulation of SDC-4 suppressed cell adhesion. (**a**) Representative inverted phase contrast microscope images of attached LECs transfected with scrambled/SDC-4 siRNA. Scale bar, 100 *μ*m. (**b**) Attached LECs were quantified by MTT assay. Data represent the mean±S.D. ***P<0.001; NS, not significant, *n*=3. (**c**) LECs were transfected with scrambled/SDC-4 siRNA and cultured on plates with/without precoating with fibronectin (Fn) for 6 h. Western blot analysis was performed to probe for p-FAK (125 kDa) and total FAK (125 kDa). (**d**) Quantification of the protein expression levels in **c**. The fold change relative to the level in the scrambled siRNA transfection group is displayed. Data represent the mean±S.D. **P<0.01, *n*=3

Integrin is presented on the surface of LECs and is an essential mediator of cell adhesion.^41^ Blockade of integrin signaling in LECs could effectively compromise LEC adhesion and prevent capsular opacification.^42^ As SDC-4 is also a co-receptor of integrin, we hypothesized that SDC-4 KD would block integrin-mediated cell adhesion. Indeed, we found reduced cell attachment to the ECM after SDC-4 KD (Figures 4a and b, the OD values for the SDC-4 siRNA group and scrambled siRNA group were 0.253±0.022 and 0.461±0.038, respectively, *P*<0.001). During cell adhesion, FAK is recruited to the cell–extracellular matrix interface and is essential for focal adhesion formation.^14^ We found that phosphorylation of FAK (p-FAK^Y397^) was significantly increased in the presence of fibronectin and was suppressed upon SDC-4 KD, while the total FAK level was not affected, indicating that SDC-4 is required for FAK activation during focal adhesion formation (Figures 4c and d).

### SDC-4 knockout suppressed injury-induced subcapsular cataract formation in an *in vivo* mouse model

**Figure 5:**
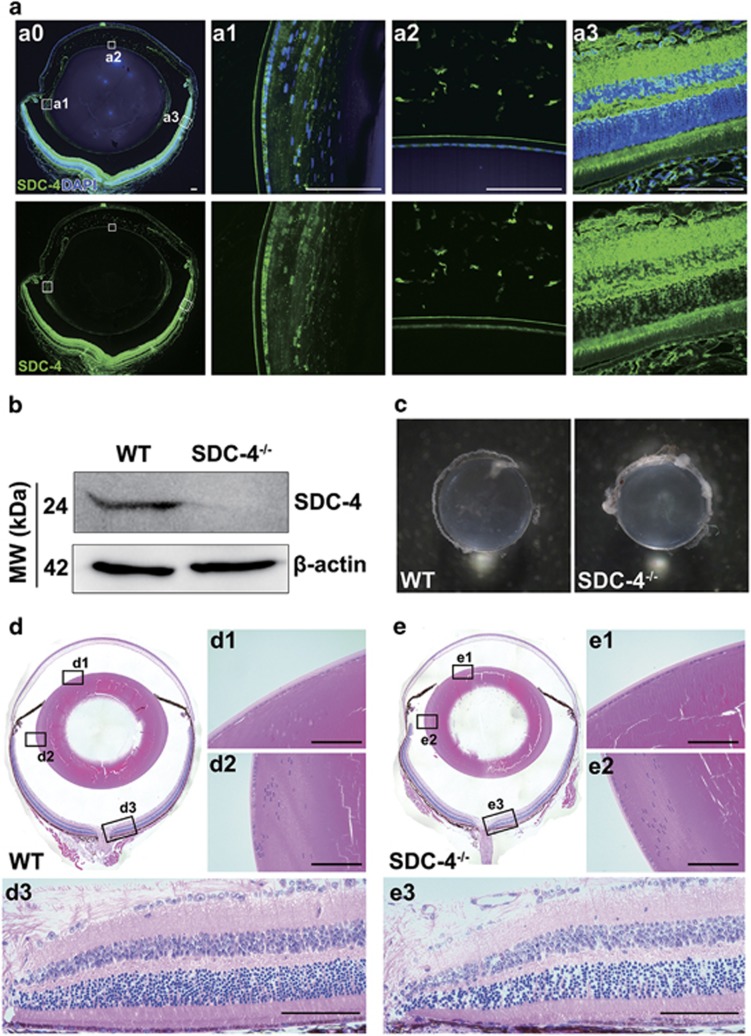
SDC-4 knockout does not affect lens development in mice. (**a**) Paraffin sections from 3-month-old WT mice were probed for SDC-4 (green). DAPI (blue) was used to stain nuclei. SDC-4 was expressed in the anterior epithelium (**a1**), lens germinative zone (**a2**), and retina (**a3**). Scale bar, 100 *μ*m. (**b**) Protein was extracted from the lenses from the 3-month-old WT and SDC-4^−/−^ mice and probed for SDC-4. (**c**) Images of the lenses isolated from the 3-month-old WT and SDC-4^−/−^ mice. (**d** and **e**) H&E staining of eyes of the 3-month-old WT (**d**) and SDC-4^−/−^ (**e**) mice. The lens anterior epithelium (**d1** and **e1**), lens germinative zone (**d2** and **e2**), and retina (**d3** and **e3**) exhibited normal histology. Scale bar, 100 *μ*m

Knowing that SDC-4 KD inhibited both FGF signaling and integrin signaling, we next examined whether SDC-4 inhibition could potentially prevent capsular opacification *in vivo*. First, we characterized the expression of SDC-4 in the eyes of 3-month-old wild-type (WT) mice, and found that SDC-4 was widely distributed in the lens epithelium, lens germinative zone, and retina (Figure 5a). The lenses from SDC-4^−/−^ mice had significantly reduced expression level of SDC-4 (Figure 5b), but were still intact and transparent as those from WT mice (Figure 5c). Hematoxylin and eosin (H&E) staining showed that lenses and retinas from SDC-4^−/−^ mice were normal as those from WT mice (Figures 5d and e). These results indicated that SDC-4 does not have a major role during the lens development. Consistently, a previous study showed that SDC-4^−/−^ mice had no macroscopic abnormalities and could reproduce normally.^43^

**Figure 6:**
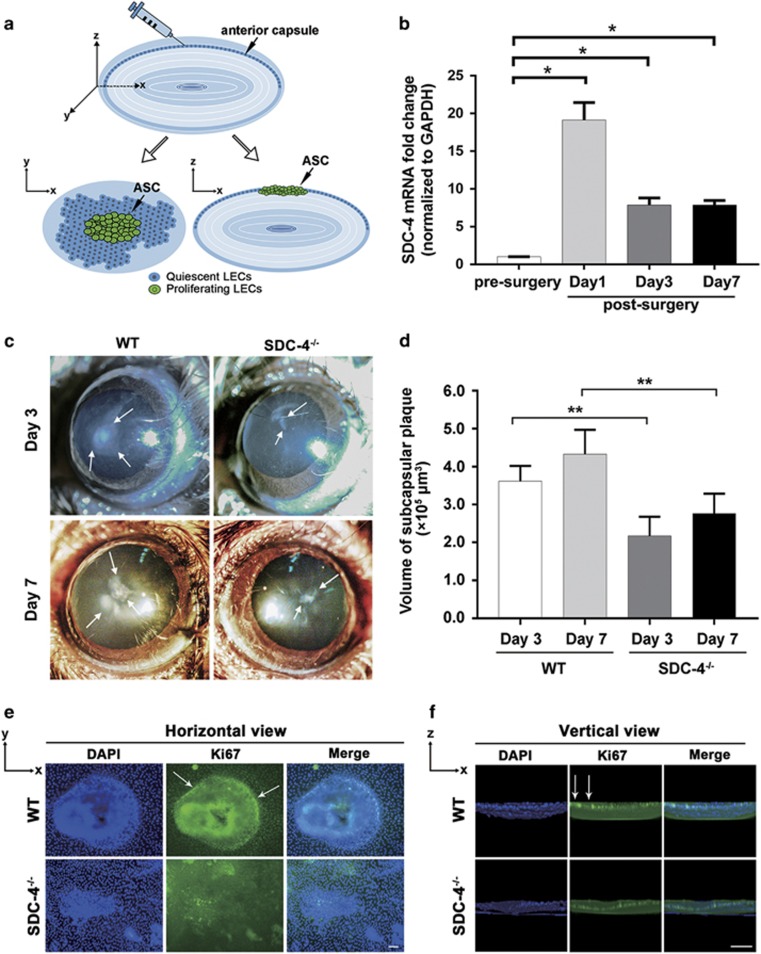
SDC-4 knockout suppressed injury-induced ASC formation in mice. (**a**) Cartoon schematic of the injury-induced ASC model. Puncture of the anterior capsule by a hypodermic needle initiates the proliferation of LECs around the injured area and finally leads to the development of ASC. (**b**) Total RNA was extracted from lenses of pre-surgery (control) or post-surgery of WT mice. The mRNA level of SDC-4 was determined using real-time PCR and normalized to GAPDH. Data represent the mean±S.D. *P<0.05, *n*=3. (**c**) Representative anterior segment slit-lamp microscope images of WT and SDC-4^−/−^ mice 3 days and 7 days after injury. White arrows indicate irregular fibrotic opacity (ASC). (**d**) Quantification of the anterior capsule opacity volume. Data represent the mean±S.D. **P<0.01, *n*=3. (**e**–**f**) Lens anterior capsule whole mounts from WT and SDC-4^−/−^ mice were stained for Ki67 (green) and DAPI (blue). White arrows indicated Ki67-positive cells. Scale bar, 50 *μ*m

**Figure 7:**
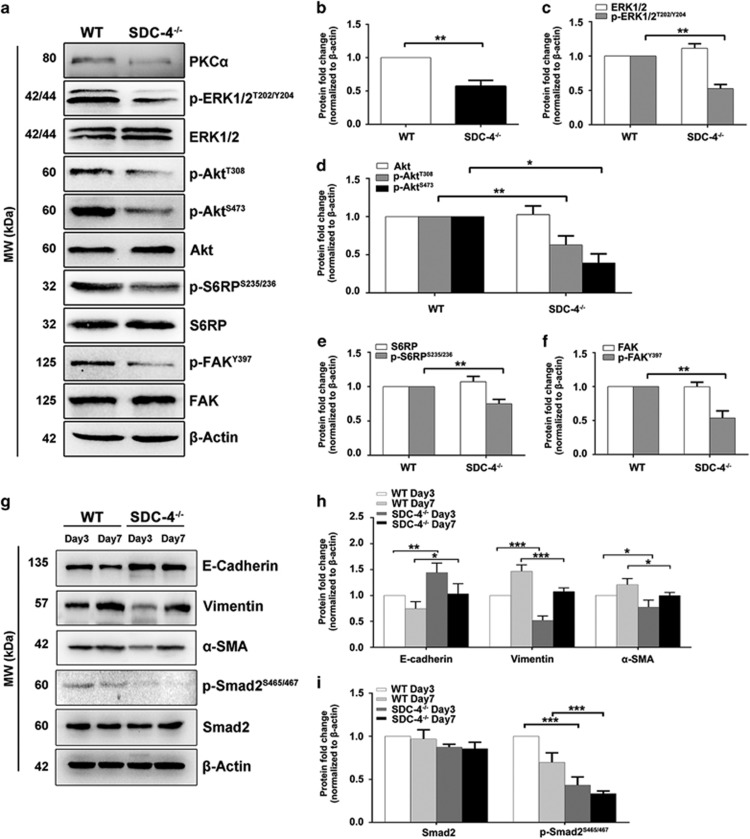
SDC-4 knockout suppressed expression of PKC*α*, inhibited activation of the FAK, ERK1/2, PI3K/Akt/mTOR, and inhibited TGF-induced EMT in the injury-induced ASC mouse model. (**a**) Protein was extracted from the lens epithelium of WT and SDC-4^−/−^ mice 3 days after injury and probed for PKC*α*, p-ERK1/2, total ERK1/2, p-Akt, total Akt, p-S6RP, total S6RP, p-FAK, and total FAK. (**b**–**f**) Quantification of the protein expression levels in **a**. The fold change relative to the level in WT mice is displayed. Protein fold change was normalized to the WT mouse group. Data represent the mean±S.D. *P<0.05, **P<0.01, *n*=3. (**g**) Protein was extracted from the lens epithelium of WT and SDC-4^−/−^ mice 3 days and 7 days after injury, and probed for E-cadherin, vimentin, *α*-SMA, p-Smad2, and total Smad2. (**h**–**i**) Quantification of the protein expression levels in **g**. The fold change relative to the level in WT mice is displayed. Protein fold change was normalized to the WT mouse group at day 3 after injury. Data represent the mean±S.D. *P<0.05, **P<0.01, ***P<0.001, *n*=3

We have previously developed an injury-induced anterior subcapsular cataract (ASC) model in which the opacity can be quantitatively analyzed.^44^ Briefly, LEC proliferation was triggered by puncturing the anterior lens capsule with a hypodermic needle (Figure 6a). Although ASC and postoperative capsular opacification are regarded as two different types of cataracts, they share common molecular and cellular mechanisms, and both are regulated by the FGF and integrin signaling pathways.^26, 27, 45, 46, 47^ First, we analyzed the expression level of SDC-4 before and after injury by real-time PCR. We found that the expression level of SDC-4 was significantly upregulated at 1 day, 3 days, and 7 days after injury (Figure 6b), which is consistent with previous findings that SDC-4 is upregulated during wound healing.^40, 48^ Next, we compared the development of ASC between wild-type (WT) mice and SDC-4-deficient (SDC-4^−/−^) mice. We found that at 3 days after injury, WT mice developed obvious anterior capsule opacities. In contrast, SDC-4^−/−^ mice only exhibited mild ASC even at day 7 after injury (Figure 6c, white arrows). Then, we used the laser scanning confocal microscopy three-dimensional (LSCM-3D) imaging to quantify the opacity. We found that the volume of the subcapsular plaque in SDC-4^−/−^ mice was significantly less than that in the WT mice at 3 days and 7 days after injury (Figure 6d). We also evaluated cell proliferation after injury through immunofluorescent staining of the injured anterior capsule whole-mount preparation by the proliferation marker Ki67. We found that SDC-4^−/−^ mice showed less Ki67-positive cells, suggesting suppressed cell proliferation after injury (Figures 6e and f). Consistent with our findings from the *in vitro* experiments, we found that the lens epithelium from SDC-4^−/−^ mice had reduced expression of PKC*α* and less phosphorylation of ERK1/2, Akt, S6RP, and FAK after injury (Figures 7a–f). Moreover, the lens epithelium from SDC-4^−/−^ mice had significant higher expression level of E-cadherin, lower expression levels of *α*-SMA and vimentin, and lower phosphorylation level of Smad2 at 3 and 7 days after injury, (Figures 7g–i) indicating suppression of transforming growth factor (TGF)-induced epithelial to mesenchymal transition (EMT). Collectively, these *in vivo* findings demonstrated that SDC-4 KO could significantly suppress LEC proliferation and capsular opacification formation.

### SDC-4 expression was increased in LECs derived from age-related cataract patients

Due to the essential role of SDC-4 during LECs proliferation, migration, and adhesion, understanding the regulation of SDC-4 in human LECs becomes key to unlocking its therapeutic potential. Although SDC-4 is widely expressed, the expression level of SDC-4 is usually low in normal tissue.^49^ Surprisingly, we found that SDC-4 mRNA expression in LECs from age-related cataract patients was approximately 7.853-fold higher than that from the human transparent lens (Supplementary Figure 6a). Consistently, immunofluorescent staining showed increased expression of SDC-4 in the LECs of the anterior capsule from age-related cataract patients (Supplementary Figure 6b). Consistent with our *in vitro* and *in vivo* results described above, findings from human patients further support the idea that deregulation of SDC-4 contributes to the development of postoperative capsular opacification, and downregulating SDC-4 may become a useful therapeutic strategy.

## Discussion

**Figure 8:**
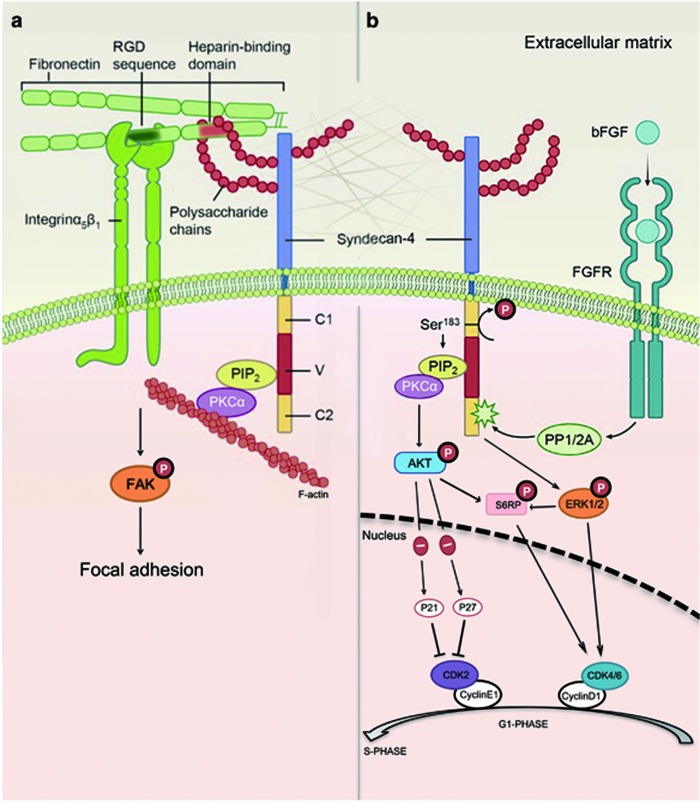
Schematic diagram of the role of SDC-4 in the regulation of the integrin and FGF signaling pathways. (**a**) SDC-4 functions as a co-receptor of integrin and mediates cell adhesion. ECM proteins, such as fibronectin, have an RGD sequence that binds to integrin and heparin-binding domains that bind to the polysaccharide side-chains of SDC-4, activating FAK and promoting focal adhesion formation. (**b**) SDC-4 also serves as a co-receptor of bFGF. The binding of FGF to FGFR activates PP1/2A, which leads to Ser^183^ dephosphorylation in the SDC-4 cytoplasmic tail. This dephosphorylation increases the binding affinity of PIP_2_ to SDC-4 and activates PKC*α*. PKC*α* further activates the PI3K/Akt/mTOR pathway. In addition, SDC-4 is required for the activation of ERK1/2, which also contributes to mTOR activation. Activation of PI3K/Akt/mTOR and ERK1/2 promotes the G1/S transition

In this study, we, for the first time, characterized the role of SDC-4 in postoperative capsular opacification formation. In FGF signaling, SDC-4 engages the PKC*α*/Akt and ERK pathways to activate mTOR, inducing cell cycle progression. In addition, SDC-4 is required for integrin-mediated cell adhesion through FAK phosphorylation (Figure 8). These results highlight the key regulatory role of SDC-4 in both FGF signaling and integrin signaling during the pathogenesis of capsular opacification. Moreover, SDC-4 inhibition suppressed FGF-induced cell cycle progression in RPE cells, indicating that SDC-4 is also critical for the development other intraocular fibrotic disorders such as proliferative vitreoretinopathy (PVR), which is resulted from similar pathological processes including proliferation, migration, and EMT of the retinal pigment epithelium.^50^

SDC-4 is distinct from other SDC family members in that its cytoplasmic tail can bind PIP_2_ and activate PKC*α*.^51, 52^ The binding of FGF to FGFR can activate a serine/threonine protein phosphatase type 1/2A (PP1/2A), which will cause Ser^183^ dephosphorylation in the SDC-4 cytoplasmic tail. This dephosphorylation increases the binding affinity of PIP_2_ to SDC-4 and activates PKC*α* (Figure 8).^20^ Indeed, we show that downregulation of SDC-4 significantly suppressed FGF-induced PKC*α* upregulation in LECs. The downstream effectors of PKC*α* may include Akt and ERK, as previously reported.^53^ Our results showed that downregulation of SDC-4 or inhibition of PKC*α* suppressed Akt^T308^ and Akt^S473^ phosphorylation, indicating that Akt is a downstream target of SDC-4 and PKC*α*. Similarly, Akt^T308^ and Akt^S473^ phosphorylation is regulated by SDC-4 in a PKC*α*-dependent manner in endothelial cells.^54, 55^ However, inhibition of PKC*α* did not affect ERK^T202/Y204^ phosphorylation, but downregulation of SDC-4 did, suggesting that ERK^T202/Y204^ activation is independent of PKC*α*, but requires SDC-4 in FGF signaling. Consistent with our findings, FGF-induced ERK phosphorylation can be inhibited through SDC-4 downregulation in human umbilical vein endothelial cells.^56^ Notably, SDC-4 facilitates FGF signaling not only through Akt and ERK activation but also via FGF internalization and nuclear localization.^38^

SDC-4 is the only member of the SDC family that is presented in focal adhesion.^57^ The formation of focal adhesion requires two signals; one is the binding of integrin to the RGD sequence on fibronectin, and the other is the binding of SDC-4 to the heparin-binding domain.^58^ Therefore, SDC-4 serves as a bridge of the cytoskeletal proteins and the ECM. In this study, we found that SDC-4 is essential for LEC adhesion to the ECM and that SDC-4 downregulation suppressed activation of FAK. This is consistent with a previous finding that SDC-4 modulates FAK tyrosine^397^ phosphorylation through a Rho-dependent manner in fibroblast cells.^59^ As FAK activation also requires PKC*α*, our finding strongly indicates that SDC-4 and PKC*α* are two key mediators that render cross-talk between FGF and integrin signaling. Indeed, FGF signaling closely interacts with integrin signaling. Rusnati *et al.*^60^ demonstrated that FGF interacts with *α*V*β*3 integrin and that blocking *α*V*β*3 integrin signaling diminishes FGF-induced adhesion and mitogenesis in endothelial cells. On the other hand, augmenting *β*1-integrin activity can restore FGF sensitivity and promote cell proliferation.^61^

We found that SDC-4 level in LECs of cataract patients is significantly upregulated, which can subsequently promote cell proliferation and migration. Notably, this is not LEC-specific, but is common in cancer cells. The expression level of SDC-4 alters significantly during tumor progression and metastasis. For example, SDC-4 expression is upregulated in testicular germ cell tumor and osteosarcoma, and is correlated with the incidence of distant metastasis.^62, 63^ SDC-4 overexpression is also highly associated with progression of breast cancer.^64^ Therefore, SDC-4 can be a potential biomarker and therapeutic target for cancer. Although specific SDC-4 inhibitor is currently unavailable, several previous studies demonstrated the efficacy of inhibitors of other SDC family members in cancer treatment. For example, nBT062, a SDC-1-specific monoclonal antibody, could inhibit the growth of multiple myeloma cells without inducing cytotoxicity against normal peripheral blood mononuclear cells.^65^ Also, OC-46F2, an antibody specifically targeting the extracellular domain of SDC-1, could inhibit tumor growth in human melanoma and ovarian carcinoma models.^66^ Moreover, an iodine-131-labeled anti-SDC-1 antibody is proved to be beneficial for refractory multiple myeloma patients in a phase I/II radioimmunotherapy study.^67^

The proliferation of the LECs and the differentiation of LECs into lens fibers are directly regulated by FGF signaling during lens development.^68, 69^ Low level of FGF was found to induce LECs proliferation, whereas higher level of FGF could promote cell cycle exit and differentiate into anuclear lens fibers.^70^ During the development of ACO and PCO, the differentiation of LECs into mesenchymal cells (EMT) is different from the differentiation of LECs into anuclear lens fibers. Tanaka *et al.*^71^ have demonstrated that during the wound healing response in lens, endogenous FGF is required for cell proliferation but not essential for EMT. Instead, TGF is the key regulator that induce LECs to undergo EMT after cataract surgery.^44, 72, 73, 74, 75^ Recently, growing evidence suggests that SDC family also participates in TGF signaling. For example, in lung adenocarcinoma A549 cells, TGF-*β*_1_ induces upregulation of SDC-4, leading to enhanced cell proliferation, migration, and EMT.^76^ Conversely, the SDC-4 KO mouse exhibited a significant reduction in aristolochic acid nephropathy-induced recruitment of TGF-*β*_1_ in the kidney and less fibrosis.^77^ We found that after injury, compared with WT mice, SDC-4 KO mice exhibited higher expression level of epithelial marker, lower expression levels of mesenchymal markers, and reduced phosphorylation of Smad2, suggesting that SDC-4 also contributes to TGF-induced EMT of LECs. The role of SDC-4 in signaling pathways of other growth factors is still open to answer in this field.

Taken together, our data from *in vitro* human LECs, *in vivo* mouse ASC model, as well as human cataract patients consistently support the idea that targeting SDC-4 can effectively suppress bFGF-induced proliferation and migration, as well as integrin-mediated adhesion of LECs. Although further translational studies are needed, our findings provide both molecular mechanisms and clinical associations as a basis for developing interventional strategies against postoperative capsular opacification through targeting SDC-4.

## Materials and Methods

### Cell culture and treatment

The immortalized human lens epithelial cell line SRA 01/04, HLE-B3 and human retinal pigment epithelial cell line ARPE-19 were kindly provided by Professor Fu Shang at Laboratory Nutrition and Vision Research (Boston, MA, USA) and cultured in Dulbecco’s Modified Eagle Medium (DMEM; GIBCO, Grand Island, NY, USA) supplemented with 10% fetal bovine serum (FBS; GIBCO, Grand Island, NY, USA) in a humidified 37 °C incubator with a 5% CO_2_ atmosphere. For siRNA transfection, the cells were plated in a six-well culture plate until they reached 70% confluence and were then transfected with SDC-4 siRNA (sc-36588, Santa Cruz Biotechnology, CA, USA) or scrambled siRNA (sc-37007, Santa Cruz Biotechnology) according to the manufacturer’s protocol. An optimal siRNA concentration of 60 nM was determined through FITC-labeled siRNA (sc-36869, Santa Cruz Biotechnology) transfection followed by flow cytometry (BD Biosciences, San Jose, CA, USA).

### Cell proliferation assay

The cells were cultured in a 96-well plate at a density of 1 × 10^4^ cells/well with serum-free medium for 24 h, transfected with siRNA, and treated with 50 ng/ml bFGF (Peprotech, Rocky Hill, NJ, USA) for 48 h. For cell counting kit-8 (CCK-8) assay, 10 *μ*l of CCK-8 solution (Dojindo, GMBH, EU) was added to each well and incubated at 37 °C for 2 h. The absorbance values were detected at a wavelength of 450 nm by a 96-well multiscanner autoreader (Synergy H1, BioTek, Winooski, VT, USA). Wells with only culture medium were used as a blank control. Inhibition rate = (OD_Scr siRNA group_−OD_SDC-4 siRNA group_)/OD_Scr siRNA group_ × 100%. Data were obtained from three independent experiments with five replicates for each group in each experiment.

### Cell cycle analysis

The cells were treated with serum-free medium for 24 h and then treated with 50 ng/ml bFGF for 48 h. Then, the cells were collected and fixed in 70% ethanol overnight at 4 °C. After that, the cells were centrifuged and suspended in 0.2 ml propidium iodide for 30 min. The cell cycle was analyzed by flow cytometry (BD Biosciences) with a 540-nm laser.

### Scratch wound assay

The cells were plated in a six-well culture plate and cultured until they reached 90% confluence. The cell monolayer was wounded by a 200-*μ*l micropipette tip and washed with phosphate-buffered saline (PBS) three times to remove cell debris. After that, the cells were cultured in FBS-free DMEM with or without 10 ng/ml bFGF for 24 h. The wound area was photographed using an inverted microscope. Image-Pro Plus 6.0 (Media Cybernetics Inc., Silver Spring, MD, USA) was used to analyze the migration distance. Cell migration index = (1−noninvading area/total wounded area) × 100%.

### Transwell migration assay

A transwell migration assay was performed using a 24-well filter plate with an 8.0-*μ*m pore size (Corning Incorporated, Corning, NY, USA). LECs in 100 *μ*l FBS-free DMEM were seeded in the upper chamber at a density of 1 × 10^5^ cells/well, and 500 *μ*l DMEM containing FGF was added to the lower chamber. After a 24 h incubation, the remaining cells in the upper side of the filter were scraped with a cotton swab, and the penetrating cells in the lower layer were fixed in methanol and stained in crystal violet. Three randomly chosen fields were photographed by using an inverted microscope. The stained cells were solubilized with 200 *μ*l 10% acetic acid, and the absorbance was measured at 600 nm by a 96-well multiscanner autoreader.

### Cell adhesion assay

The cells treated with siRNA for 48 h were trypsinized, resuspended, and added to a 96-well plate precoated with fibronectin (sc-29011, Santa Cruz Biotechnology) at a density of 1 × 10^4^ cells/well. After incubation at 37 °C for 3 h, the cells were washed gently with PBS three times. Cell adhesion was photographed by using an inverted microscope. Then, 10 *μ*l of 5 mg/ml MTT was added to each well and incubated at 37 °C in a CO_2_ incubator for 4 h. The formazan granules were dissolved in 150 *μ*l DMSO, and the absorbance values were detected at a wavelength of 490 nm by a 96-well multiscanner autoreader.

### Mouse lens capsular injury model

SDC-4 knockout (KO) mice were kindly provided by Professor Rong Ju at Zhongshan Ophthalmic Center (Guangzhou, Guangdong, China).^78, 79^ Six- to eight-week-old SDC-4^−/−^ and age-matched WT mice were used in the study. All animal experiments were conducted in accordance with the approved guidelines of the Ethics Committee in Animal Experimentation of Zhongshan Ophthalmic Center (No. 2016-015). Mouse lens capsular injury was performed as described previously.^44^ Briefly, the mice were anesthetized with 0.2 ml 5% Cholrali Hydras and dicaine eyedrop. After topical application of mydriatic, a 26-gauge hypodermic needle was used to make an incision in the central anterior capsule of the right eye through the cornea. The depth of the incision was one-fourth the length of the blade (~300 *μ*m). The mice were photographed by using a slit lamp 3 days after injury.

### Collection of human anterior capsule samples

The anterior capsule samples were obtained from aged-related cataract patients during cataract surgeries. All the surgeries were conducted by one surgeon (Mingxing Wu), and each capsule was about 5 mm in diameter. Informed consent was obtained from the patients before surgery. Age-matched anterior capsule samples from transparent lens were obtained from cadaver eye in the eye bank of Zhongshan Ophthalmic Center. The research conformed to the tenets of the Declaration of Helsinki and followed the protocol approved by the Institutional Research Board of Zhongshan Ophthalmic Center. Lens anterior capsule whole mounts were prepared as previously described.^26^

### Immunofluorescence

For immunofluorescent staining of the whole-mount lens anterior capsules in the capsular injury model, the mice were killed, and the eyes were enucleated 3 days after injury. The anterior capsules were isolated under a dissecting microscope. The capsules were immediately fixed in 100% methanol for 1 h at room temperature (RT), permeated with 0.1% Triton X-100 for 30 min, and blocked with 1% bovine serum albumin (BSA) for 60 min. Then, the capsules were incubated with the anti-Ki67 antibody (1:250; Abcam, Cambridge, MA, USA) diluted in 1% BSA at 4 °C overnight, briefly washed with PBS containing 0.1% Tween 20 (PBST), and incubated with the FITC-conjugated antibody (1:500; Cell Signaling Technology, Danvers, MA, USA) at RT for 60 min. The nuclei of the cells were stained with DAPI for 5 min. The capsule was placed flat on a microscope slide under a dissecting microscope and covered with a coverslip. Images were observed by using a Zeiss LSM 510 confocal microscope. Two-dimensional (2D) images were taken at 2 *μ*m intervals to generate z-stacks and then converted into three-dimensional (3D) images. The volume of the anterior capsule opacities between two images was calculated using the formula of a frustum 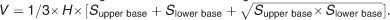 The basal area was measured by using Image-Pro Plus 6.0 (Media Cybernetics Inc.). The total volume of the anterior capsule plaque was calculated as *V*_total_=*V*_1_+*V*_2_+…+*V_n_*.

For immunofluorescent staining of the paraffin section, eyes were enucleated from killed 3-month-old mice, fixed with 4% paraformaldehyde for 24 h, dehydrated in ethanol, and embedded in paraffin. Paraffin sections with 4 *μ*m thickness were incubated at 60 °C for 30 min and dewaxed using xylenes. After dehydrated in ethanol and antigen retrieval, sections were subject to immunohistochemical staining. Images were taken by Zeiss LSM 510 confocal microscope.

For immunofluorescent staining of the cultured LECs/anterior capsule whole mounts, the samples were fixed with 4% paraformaldehyde, permeabilized with 0.1% Triton X-100, blocked with 1% bovine serum albumin (BSA) for 60 min. After that, the samples were incubated with the anti-SDC-4 antibody (1:100; Abcam) at 4 °C overnight and FITC-conjugated antibody (1:500; Cell Signaling Technology) at RT for 60 min. The nuclei of the LECs were stained with DAPI for 5 min. Images were taken by using a Zeiss LSM 510 confocal microscope.

### H&E staining

The 3-month-old WT mice and SDC-4^−/−^ mice were killed. The eyes were enucleated and fixed with 4% paraformaldehyde for 24 h at room temperature. The paraffin sections were sectioned from the optic nerve at 4 *μ*m thickness, and stained with H&E. Images were taken by a Leica DM4000 B upright microscope.

### Western blot analysis

The cells or lens tissue were lysed in RIPA buffer supplied with protease and phosphatase inhibitor cocktail. The protein concentration was determined using the BCA-100 Protein Quantitative Analysis Kit (Biocolor Bioscience & Technology Co., Ltd., Shanghai, China). Equal amounts of protein (15 *μ*g/lane) were resolved in 10% sodium dodecyl sulfate-polyacrylamide gel electrophoresis (SDS-PAGE). The proteins were then transferred to polyvinylidene fluoride membranes (PVDF membranes; Millipore, MA, USA) for probing with primary antibodies and horseradish peroxidase (HRP)-conjugated secondary antibodies. Signals were detected with an eECL Western Blot Kit (ComWin Biotech Co., Ltd, Beijing, China). *β*-actin was used as a reference. The sources of the antibodies used are as follows: rabbit anti-SDC-4 (1:1000; Abcam), mouse anti-cyclin E1 (1:1000; Cell Signaling Technology), mouse anti-cyclin D1 (1:2000; Cell Signaling Technology), mouse anti-CDK6 (1:1000; Cell Signaling Technology), mouse anti-CDK4 (1:1000; Cell Signaling Technology), rabbit anti-CDK2 (1:1000; Cell Signaling Technology), rabbit anti-P27 (1:1000; Cell Signaling Technology), rabbit anti-P21 (1:2000; Cell Signaling Technology), mouse anti-PCNA (1:1000; Novus Biologicals, Littleton, CO, USA), rabbit anti-caspase-3 (1:1000; Abcam), rabbit anti-caspase-9 (1:1000; Abcam), rabbit anti-PKC*α* (1:1000; Cell Signaling Technology), rabbit anti-p-Akt (1:500; T308, Cell Signaling Technology), mouse anti-p-Akt 1:500; S473, Cell Signaling Technology), rabbit anti-Akt (1:1000; Cell Signaling Technology), rabbit anti-p-ERK1/2 (1:1000; T202/Y204, Cell Signaling Technology), rabbit anti-ERK1/2 (1:1000; Cell Signaling Technology), rabbit anti-p-S6RP (1:2000; S235/236, Cell Signaling Technology), rabbit anti-S6RP (1:1000; Cell Signaling Technology), rabbit anti-p-FAK (1:500; Y397, Cell Signaling Technology), rabbit anti-FAK (1:1000; Cell Signaling Technology), rabbit anti-E-cadherin (1:1000; Cell Signaling Technology), mouse anti-vimentin (1:1000; Abcam), mouse anti-*α*-SMA (1:1000; Abcam), rabbit anti-p-Smad2 (1:1000; S465/467, Cell Signaling Technology), rabbit anti-Smad2 (1:1000; Cell Signaling Technology) and mouse anti-*β*-actin (1:2000; Cell Signaling Technology). The signal density was quantified by using ImageJ 1.48 (National Institutes of Health, Bethesda, MD, USA).

### Total RNA extraction and real-time PCR

Total RNA was isolated from tissue samples and LECs using RNEasy Micro kit (Qiagen, Hilden, Germany) and high pure RNA tissue kit (Roche, Mannheim, Germany), respectively. A Transcriptor First Strand cDNA synthesis kit (Roche) was used for reverse transcription. The LightCycler 480 SYBR Green I Master (Roche) was used to amplify the target genes, which were detected by using a LightCycler480II real-time PCR system (Roche). *β*-actin and GAPDH were used as an internal control. Primer sequences were as follows: human SDC-4 (F), 5′-CTCCTAGAAGGCCGATACTTCT-3′ human SDC-4 (R), 5′-GGACCTCCGTTCTCTCAAAGAT-3′ human *β*-actin (F), 5′-GGACTTCGAGCAAGAGATGG-3′ human *β*-actin (R), 5′-AGCACTGTGTTGGCGTACAG-3′ mouse SDC-4 (F), 5′-ATGTCCAACAAAGTATCCATGTCCA-3′ mouse SDC-4 (R), 5′-ATGCGGTACACCAGCAGCAG-3′ mouse GAPDH (F), 5′-GCCAAGGCTGTGGGCAAGGT-3′ mouse GAPDH (R), 5′-TCTCCAGGCGGCACGTCAGA-3′.

### Statistical analysis

Statistical analysis was performed with a statistical software package (SPSS, version 16.0, Chicago, IL, USA). The data of each group were compared and expressed as the mean±S.D. Statistical significance was determined by independent sample *t*-test between two groups or by one-way analysis of variance (ANOVA) followed by *post hoc* Fisher's least significant difference (LSD) test between multiple groups. All tests were two-tailed. Values of *P*<0.05 were considered statistically significant.
