# Supplementary material for: Killing two birds with one stone: dual blockade of integrin and FGF signaling through targeting syndecan-4 in postoperative capsular opacification
Source: Cell Death Dis. 2017 Jul 13;8(7):e2920–. doi: 10.1038/cddis.2017.315 (PMC5550862; doi:10.1038/cddis.2017.315)
Supplement: Supplementary Information [file cddis2017315x1.docx]

**Supplemental information summary**

1. Supplemental information summary and supplementary figure legends (Supplementary information.docx)

2. Supplemental figures (Fig S1-S6)

**Supplementary figure legends**

**Supplementary Figure 1.** Downregulation of SDC-4 mRNA after RNA interference. (**a**) LECs were transfected with scrambled/SDC-4 siRNA for 48 h. Then cells were probed for SDC-4 (green). DAPI (blue) was used to stain nuclei. Scale bar: 20 μm. (**b**) Proteins were extracted from cells and probed for SDC-4 (24 kDa). β-actin (42 kDa) was used as an internal control. (**c**) The mRNA level of SDC-4 was determined by real-time PCR and normalized to the β-actin level. Data represent the mean ± S.D. ****P*<0.001, NS: not significant, n=3.

**Supplementary Figure 2.** Downregulation of SDC-4 suppressed FGF-induced cell proliferation. LECs were transfected with scrambled/SDC-4 siRNA for 48 h. Cell proliferation was analyzed after 50 ng/ml bFGF treatment for 48 h. The OD values at 48 h after treatment were divided by the OD values at 12 h after treatment, and normalized to the untransfected group. Data represent the mean ± S.D. **P*<0.05, ***P*<0.01, n=3.

**Supplementary Figure 3.** Downregulation of SDC-4 did not increase cell apoptosis. LECs were transfected with scrambled/SDC-4 siRNA and treated with/without 50 ng/ml bFGF for 48 h. Western blot analysis was performed to probe for pro-caspase-3 (32 kDa), cleaved caspase-3 products (17 kDa, 19 kDa) (**a**) or pro-caspase-9 (46 kDa), cleaved caspase-9 products (35 kDa, 37 kDa, 39 kDa) (**b**) as apoptosis markers.

**Supplementary Figure 4.** Downregulation of SDC-4 suppressed FGF-induced proliferation of HLE-B3 cells. (**a**) HLE-B3 cells were transfected with scrambled/SDC-4 siRNA for 48 h. Cell proliferation was analyzed by CCK-8 assay after 50 ng/ml bFGF treatment for 48 h. Data represent the mean ± S.D. ***P*<0.01, ****P*<0.001, n=3. (**b**) LECs were transfected with scrambled/SDC-4 siRNA and treated with/without 50 ng/ml bFGF for 48 h. Western blot analysis was performed to probe for PCNA (30 kDa), cyclin E1 (56 kDa), cyclin D1 (36 kDa) and P21 (21 kDa). (**c**) Quantification of the protein expression levels in **b**. The fold change relative to the level in the untransfected group is displayed. Data represent the mean ± S.D. **P*<0.05, ***P*<0.01, ****P*<0.001, n=3.

**Supplementary Figure 5.** Downregulation of SDC-4 suppressed FGF-induced cell proliferation of ARPE-19 cells. (**a**) ARPE-19 cells were transfected with scrambled/SDC-4 siRNA for 48 h. Cell proliferation was analyzed by CCK-8 assay after 50 ng/ml bFGF treatment for 48 h. Data represent the mean ± S.D. ***P*<0.01, n=3. (**b**) Cells were transfected with scrambled/SDC-4 siRNA and treated with/without 50 ng/ml bFGF for 48 h. Western blot analysis was performed to probe for PCNA (30 kDa), cyclin D1 (36 kDa) and P21 (21 kDa). (**c**) Quantification of the protein expression levels in **b**. The fold change relative to the level in the untransfected group is displayed. Data represent the mean ± S.D. **P*<0.05, ***P*<0.01, n=3.

**Supplemental Figure 6.** SDC-4 expression level is increased in the anterior capsule LECs of cataract patients. (**a**) Total RNA was extracted from the anterior capsules of age-related cataract patients and age-matched postmortem human lens (control). The mRNA level of SDC-4 was determined using real-time PCR and normalized to β-actin. Data represent the mean ± S.D. ***P*<0.01, n=4. (**b**) Lens anterior capsule whole-mounts from age-related cataract patients and age-matched postmortem human lens (control) were probed for SDC-4 (green). DAPI (blue) was used to stain nuclei. Images were acquired from the central area of each sample. Scale bar: 10 μm.
