# Supplementary figures and images for: Killing two birds with one stone: dual blockade of integrin and FGF signaling through targeting syndecan-4 in postoperative capsular opacification
Source: Cell Death Dis. 2017 Jul 13;8(7):e2920–. doi: 10.1038/cddis.2017.315 (PMC5550862; doi:10.1038/cddis.2017.315)

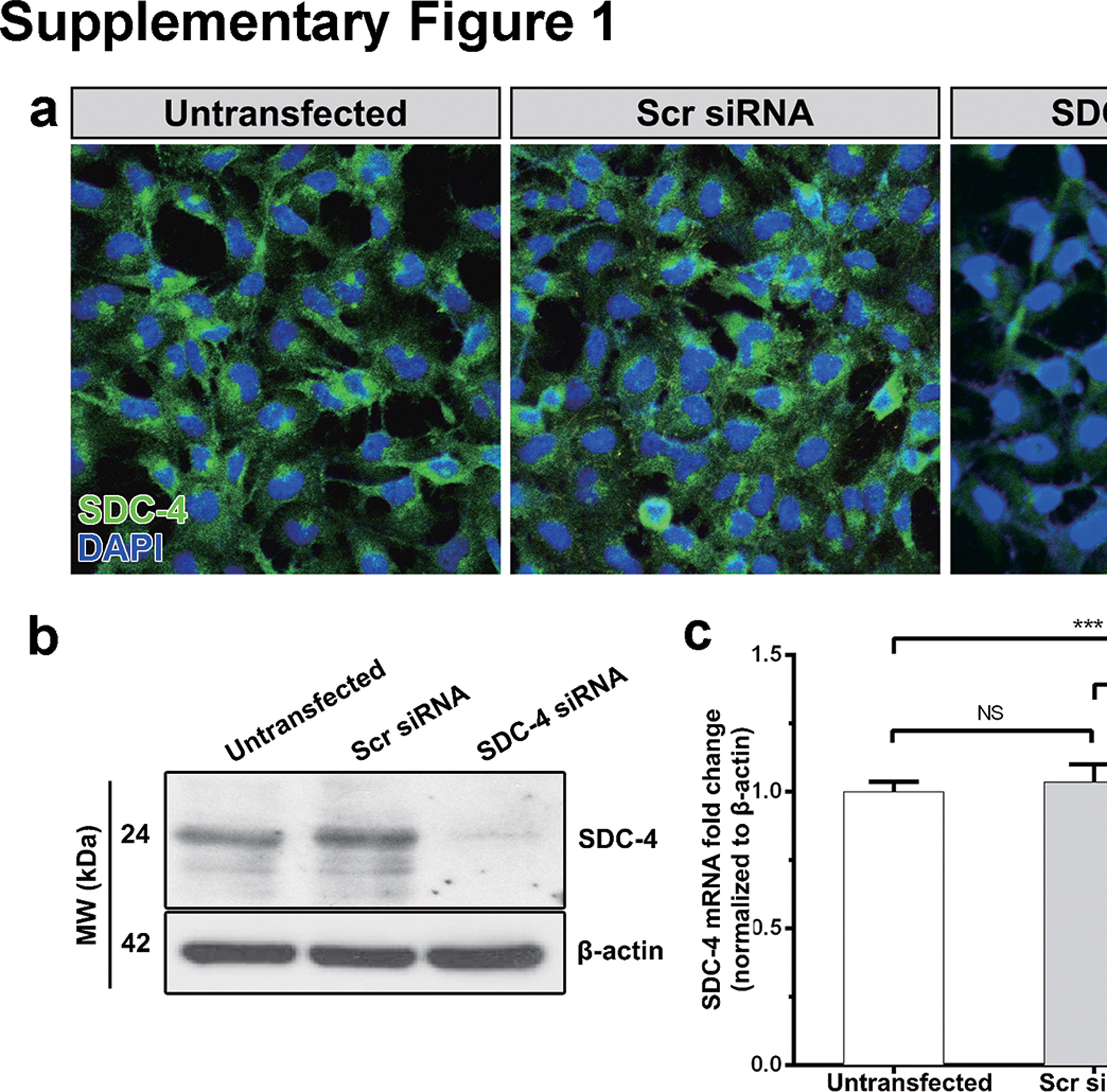

Supplement: Supplementary Figure 1 [file cddis2017315x2.tif]

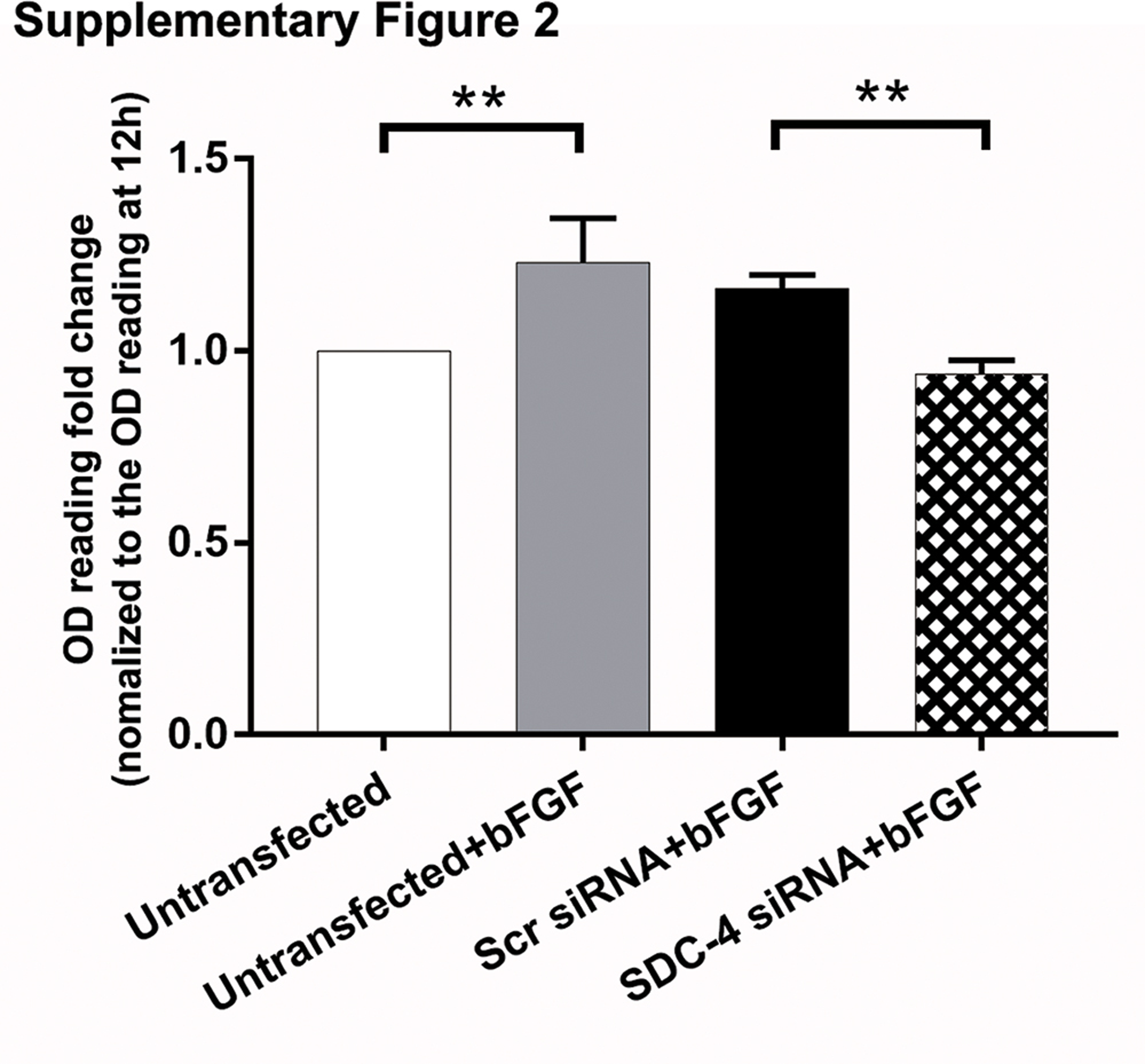

Supplement: Supplementary Figure 2 [file cddis2017315x3.tif]

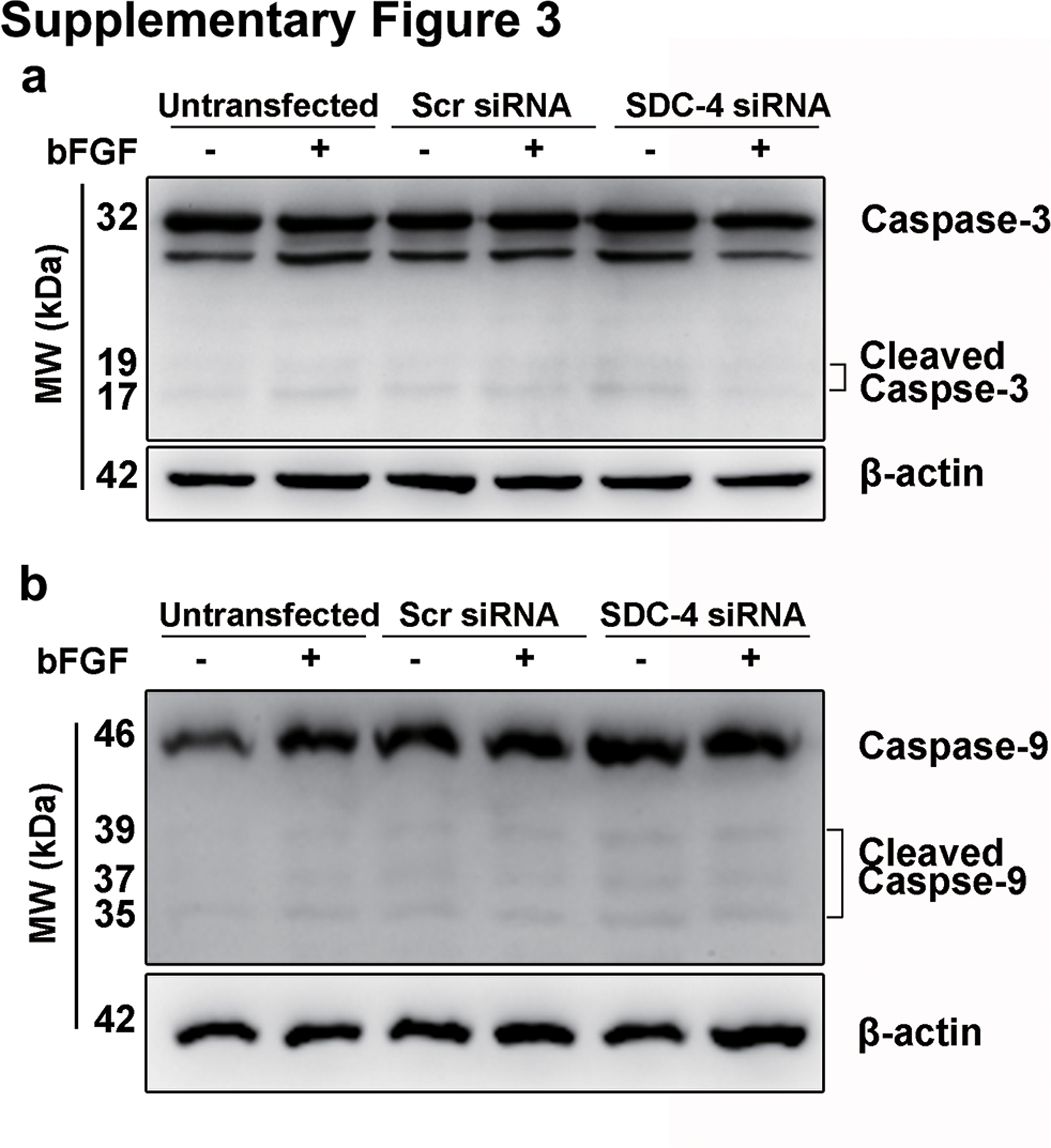

Supplement: Supplementary Figure 3 [file cddis2017315x4.tif]

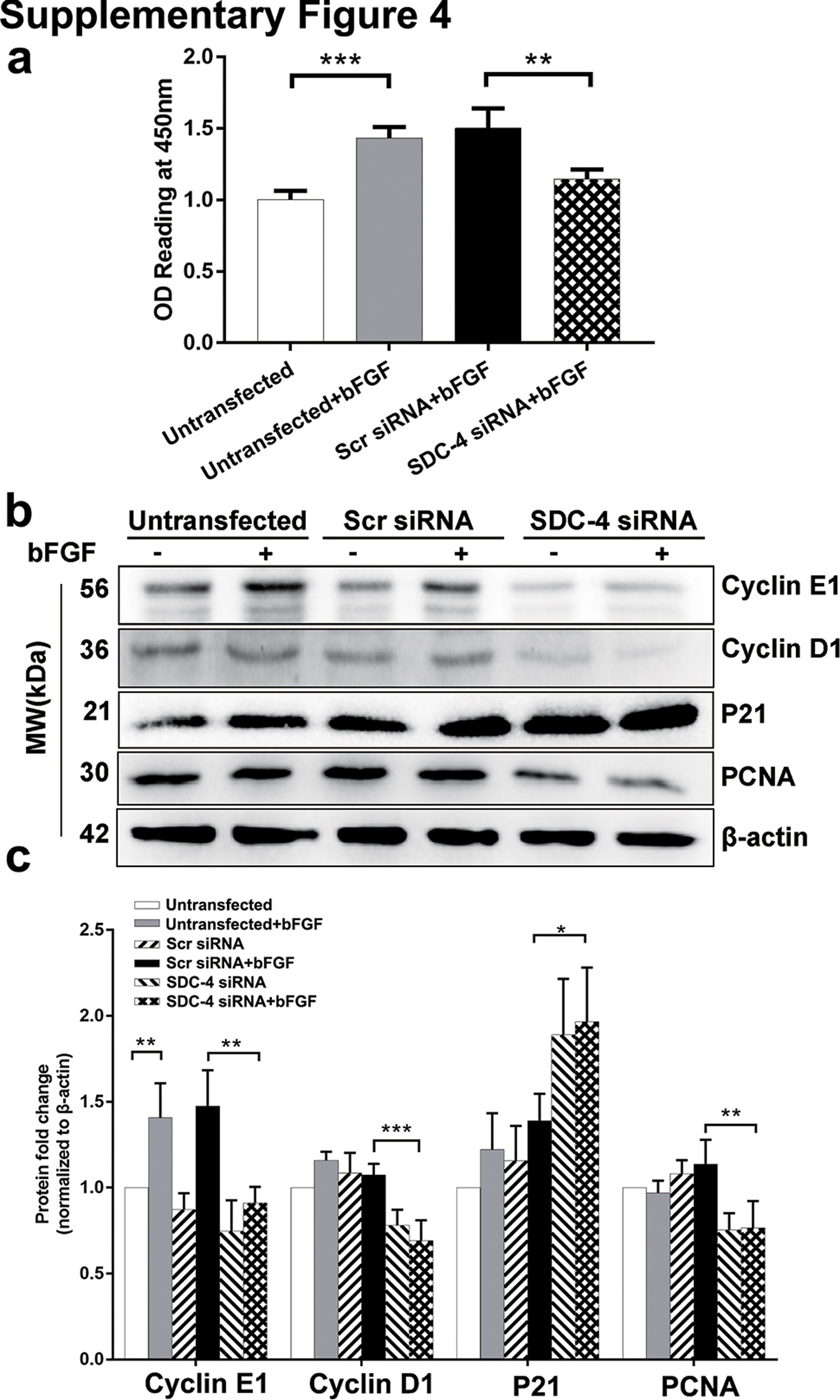

Supplement: Supplementary Figure 4 [file cddis2017315x5.tif]

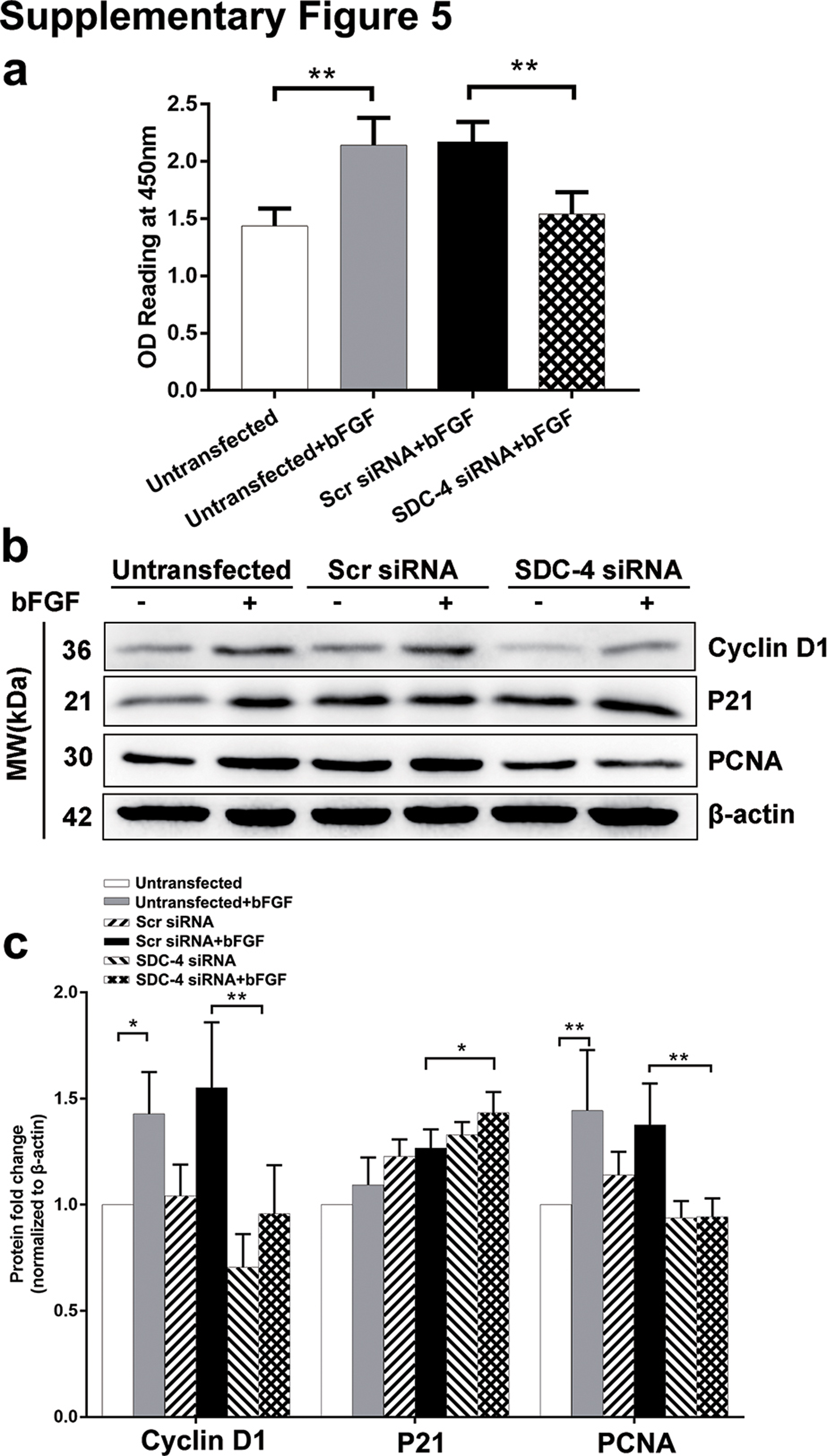

Supplement: Supplementary Figure 5 [file cddis2017315x6.tif]

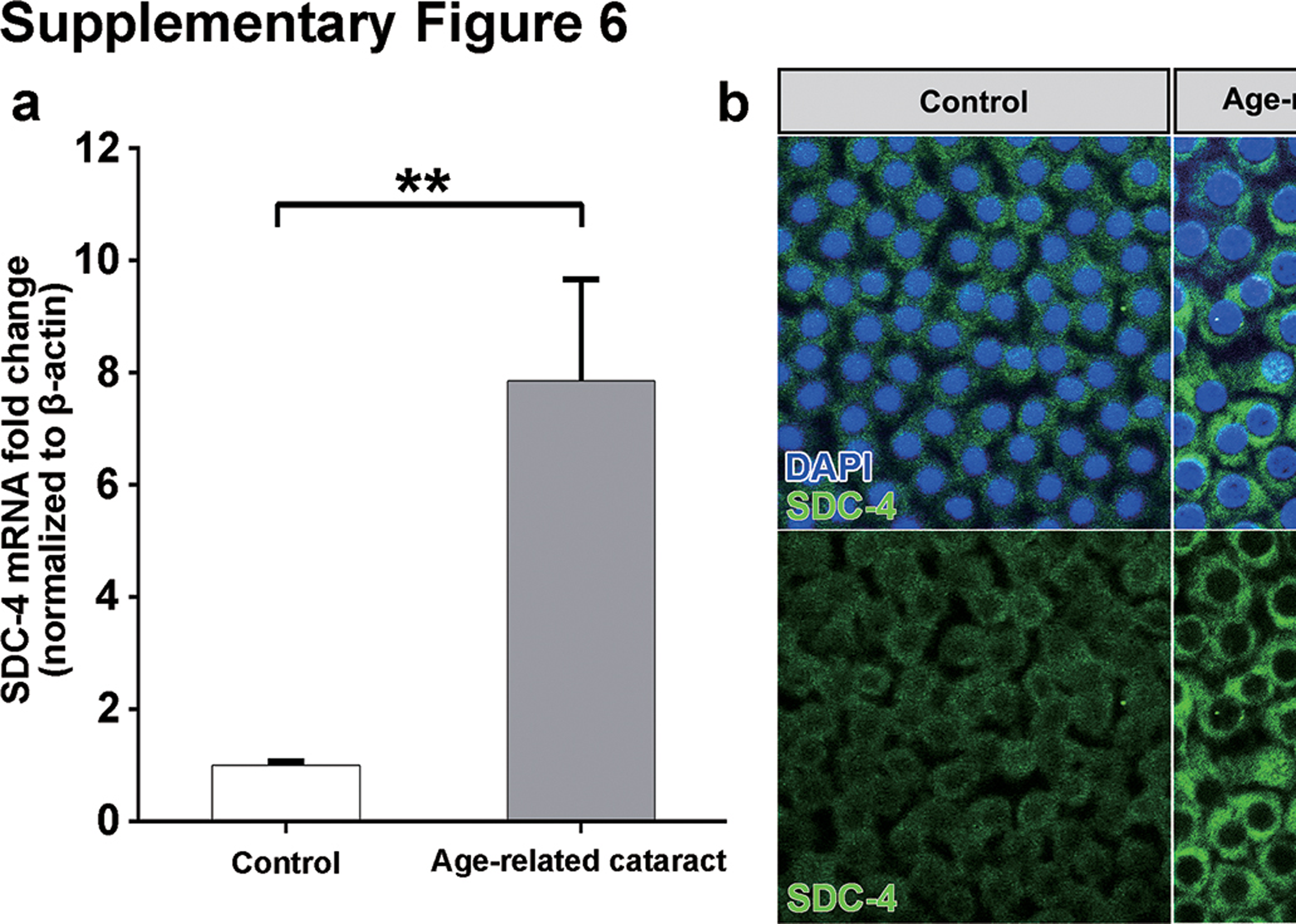

Supplement: Supplementary Figure 6 [file cddis2017315x7.tif]
